# Circular RNAs: Potential Regulators of Treatment Resistance in Human Cancers

**DOI:** 10.3389/fgene.2019.01369

**Published:** 2020-01-28

**Authors:** Shivapriya Jeyaraman, Ezanee Azlina Mohamad Hanif, Nurul Syakima Ab Mutalib, Rahman Jamal, Nadiah Abu

**Affiliations:** UKM Medical Molecular Biology Institute (UMBI), UKM Medical Center, Kuala Lumpur, Malaysia

**Keywords:** non-coding RNAs, biomarker, RNA splicing, chemoresistance, radioresistance

## Abstract

Circular RNAs (circRNAs) which were once considered as “junk” are now in the spotlight as a potential player in regulating human diseases, especially cancer. With the development of high throughput technologies in recent years, the full potential of circRNAs is being uncovered. CircRNAs possess some unique characteristics and advantageous properties that could benefit medical research and clinical applications. CircRNAs are stable with covalently closed loops that are resistant to ribonucleases, have disease stage-specific expressions and are selectively abundant in different types of tissues. Interestingly, the presence of circRNAs in different types of treatment resistance in human cancers was recently observed with the involvement of a few key pathways. The activation of certain pathways by circRNAs may give new insights to treatment resistance management. The potential usage of circRNAs from this aspect is very much in its infancy stage and has not been fully validated. This mini-review attempts to highlight the possible role of circRNAs as regulators of treatment resistance in human cancers based on its intersection molecules and cancer-related regulatory networks.

## Introduction

Increasing evidence has shown that circular RNAs (circRNAs) a form of non-coding RNA is involved in important biological processes and cellular functions (Kristensen et al., 2018). Though the concept of circRNAs first emerged in 1976, it became particularly interesting in recent years when it was found to have regulatory roles in cancer biology. During the early years of discovery, the occurrence of circRNAs was considered as post-transcriptional errors; however, with the advent of various biomedical technologies, the biogenesis and functions of circRNAs have been extensively explored (Wang et al., 2017). As of 2018, there were approximately 30000 circRNAs identified and the numbers are increasing (Si-Tu et al., 2019). There are four categories of circRNAs, exonic circRNA (ecircRNA), circular intronic RNA (ciRNA), exon-intron circRNAs, and intergenic circRNAs (Li Z. et al., 2015; Memczak et al., 2013; Zhang et al., 2013). CircRNAs have covalently closed loops without a free 3′ or 5′ ends (Lasda and Parker, 2014). It is postulated that the formation of circRNAs are *via* back-splicing where the downstream exons are spliced to upstream exons in reverse order in the primary transcript (Chen and Yang, 2015). Furthermore, several unique properties make circRNAs a promising entity in providing key insights into human diseases. Besides being abundant both in normal and cancer cells, it was also found that circRNAs are specifically expressed at every stage of cell development (Li J. et al., 2015). It was further confirmed that different isoforms of circRNAs from the same gene are expressed differently in different cell types. In several types of cancers such as hepatocellular carcinoma and colorectal cancer, it was noted that the expression level of circRNAs varies according to TNM stage, presence of metastasis and size of tumor (Szabo and Salzman, 2016). Unlike linear RNAs, circRNAs are more stable and are not easily degraded by ribonucleases such as exonuclease or RNase R due to the unexposed 3′ and 5′ terminals (Wang et al., 2017). Moreover, most circRNAs have an average half-life of over 48 h compared to linear mRNA with an average half-life of 10 h, thus making it more available for both research and clinical purposes. In addition to its advantageous properties, studies have found that circRNAs are involved in several biological activities as competing endogenous RNA by sponging miRNAs (Lin ADF and Chen, 2018), RNA binding proteins (RBPs) (Wang et al., 2015) and translating peptides (Granados-Riveron and Aquino-Jarquin, 2016; Du et al., 2017). Of particular interest is the role of circRNAs as miRNA sponge in tumor pathogenesis, and there have been many publications related to this (Wang et al., 2015; Zhang et al., 2017; Kun-Peng et al., 2018). By serving as a miRNA sponge with many binding sites, circRNAs can regulate the expression of miRNA as a competitive inhibitor that suppresses the ability of the miRNA to bind to its target genes. This event can, in turn, increase the levels of the miRNA target causing dysregulation of gene expression and pathological effects on tumor environment (Huang et al., 2015; Palmieri et al., 2018; Zeng et al., 2018). Some of these potential miRNA targets have been reported to function as important regulators of various cellular processes including apoptosis, invasion, migration, and drug resistance in several cancers.

Recently, much evidence was published on the role of circRNAs in disease progression and activation of key pathways like EMT and Wnt (Shen et al., 2019; Wu et al., 2019). Cancers that are gaining popularity like gastric, hepatocellular, lung, and breast are being studied closely with the hope to target the specific circRNAs that are involved in the development of tumor (Shang et al., 2019). Accumulating data on the association between circRNA and tumorigenesis shows promising results. However, little is known about its role in cancer therapy resistance. As therapy resistance remains one of the major clinical hurdles in cancer management, this mini-review aims to explore the potential of circRNAs as a regulator of treatment resistance. We reviewed recent relevant publications focusing on circRNAs in treatment resistance, particularly regarding drug therapy and radiotherapy. We also looked at studies at the network level to explain the relationship of circRNAs with the potential targets and pathways that could influence disease progression.

### CircRNA Effects Radiotherapy Receptivity *via* WNT Pathway

Non-coding RNAs have been linked to tumorigenesis, metastasis, and the development of resistance to treatment (Gong et al., 2014). Radiation therapy is one of the main treatment solutions for esophageal squamous cell carcinoma (ESCC) patients, especially with unresectable esophageal cancer. Unfortunately, radioresistance has been one of the reasons for failed treatments and local tumor recurrence in ESCC (Chen et al., 2017). In a study conducted by Su et al, hsa_circ_001059 and hsa_circ_000167 levels were shown to be dysregulated in radioresistant ESCC cell line as compared to the parental cell line (Su et al., 2016). The analysis showed that circRNA_001059 could sponge to multiple miRNAs including miR-30c-1, miR-30c-2, miR-122, miR-139-3p, miR-339-5p, and miR-1912. In support of this finding, miR-30 and miR-122 were found to be dysregulated in chemoresistant prostate cancer and miR-30 in radiosensitive leukemia cells (Ni et al., 2017; Liamina et al., 2017). These dysregulated circRNAs were mapped to their target genes and were found to be mainly involved in the Wnt signaling pathway and the PI3K/Akt pathway. The crosstalk mechanisms between the Wnt pathway and other non-coding RNAs such as the long non-coding RNA (lncRNAs) have been observed in cancer pathogenesis (Yang et al., 2018) including in breast cancer (Liu et al., 2016; Koval and Katanaev, 2018) and lung cancer (Wan et al., 2016; Zeng et al., 2017). Wnt signaling pathway was shown to be an important regulator in cancer cell responsiveness to radiotherapy (Takebe et al., 2015). The activation of the pathway increases the rate of DNA double-strand break repair and is also involved in breast cancer and colorectal cancer chemotherapy resistance (Flanagan et al., 2015; Pohl et al., 2017). Moreover, Li et al. have found that the expression of circITCH was low in ESCC tissues. It is possible that circITCH could influence the radioresistance of ESCC by inhibiting the target gene *via* suppression of the Wnt/β signaling pathway as in a previous radioresistance study (Li J. et al., 2015). Besides ESCC, circITCH has also been associated with other cancers as well such as hepatocellular, bladder, lung, and colorectal where it acts as miRNA sponges particularly to miR-7, miR-17 and miR-214 (Li J. et al., 2015; Guo et al., 2018; Shang et al., 2019). Due to the sponging effect, the expression of the protein-coding gene ITCH increases as well (Fang, 2018). It was discovered that the ITCH gene is an important player in tumor formation and responsiveness to chemotherapy (Li J. et al., 2015). Phosphorylation of the mediator protein Dvl2 is required for activation of Wnt signaling and previous research have found that ITCH gene can disrupt Dvl2 phosphorylation and subsequently lead to the inhibition of the signaling pathway (Li J. et al., 2015). It has also been shown that parallel circITCH expression level increases with *ITCH* mRNA level in colorectal cancer (Huang et al., 2015). Sponging effects of circITCH to miR-7 and miR-20a lead to the downregulation of *ITCH via* bindings of these miRNAs to the 3'UTR of *ITCH*, ultimately attenuating the proliferative rate of CRC cell lines (Huang et al., 2015).

### Multiple CircRNAs Regulate Chemoresistance *via* MAPK Pathway

Chemoresistance poses as one of the biggest challenges for cancer patients receiving neoadjuvant, adjuvant or palliative chemotherapy. Similar to radioresistance, the possibility of circRNAs as chemoresistance regulators were recently explored. Evidence on this is still limited but seems promising. It is well known that the Mitogen-Activated Protein Kinase (MAPK) signaling pathway is dysregulated in many cancers such as breast, pancreatic, colon, and leukemia (Li et al., 2014). MAPK pathway is important in controlling cellular activities including but not limited to proliferation, differentiation, apoptosis, survival, inflammation, and influence gene expression. This pathway has also shown potential in regulating treatment resistance involving non-coding RNAs in non-small cell lung cancer and malignant melanoma (Li et al., 2014). A study on EGFR therapy in colorectal cancer showed that global circRNAs were significantly downregulated in mutant Kras cell line, particularly circFAT1 and circARHGAP5 (Dou et al., 2016). Kras functions in activating the MAP3K tier which in turn activates the ERK1/2 cascade while EGFR is an important growth factor which can act as an oncogene by hyper-activating the signaling pathway (Hymowitz and Malek, 2018). In line with this observation, some studies showed that overexpression of EGFR can promote radio and chemotherapy resistance by activating the second and third tiers of the MAPK pathway (Pozzi et al., 2016). The MEK/ERK cascade was shown to be exclusively deactivated when EGFR expression is inhibited. These findings suggest that dysregulation in any tier of this cascade affects the levels of circRNA and may contribute to treatment resistance. However, it is difficult to conclude from this study that the lower levels of circRNA is a regulator of the oncogenic factors or it directly protects the cancer cells from chemoradiation treatment. Thus, a further functional investigation is required to define the role of circRNAs in this instance. Dysregulation of the MAPK pathway was also found in lung adenocarcinoma (LUAD) treatment resistance whereby the increased level of circRNA CCDC66 contributes to the overexpression of EGFR (Joseph et al., 2018). CircCCDC66 acts as a sponge for multiple miRNAs and therefore can have a wider effect on its targeted genes (Weng et al., 2017). Besides the MAPK signaling pathway, HGF-MET pathway was also reported to be involved. The role of HGF-MET pathway in EGFR targeted therapy resistance was first reported in 2007 and since then many studies have shown that increase in its activity causes an elevation in RAS-RAF-MEK-ERK or the MAPK pathways (Ko et al., 2017). Thus, current studies are trying to approach the dual inhibition of MET and EGFR in managing resistance in LUAD patients (Ko et al., 2017). Another notable circRNA that plays a role in MAPK pathway is ciRS-7, which is known to sponge to miR-7 and elevates the expression of target genes (Hansen et al., 2013a; Weng et al., 2017). ciRS-7 is known as a super sponge for miR-7 with more than 70 binding sites and act as an expression inhibitor (Hansen et al., 2013a; Hansen et al., 2013b). MiR-7 which is a well-researched miRNA is known to be a player in the progression of many types of cancers in human and directly targets key oncogenes such as EFGR (Kefas et al., 2008), c-KIT in brain cancer (Tamim et al., 2014), PAX6 in colorectal cancer (Needhamsen et al., 2014) and AKT in hepatocellular carcinoma (Fang et al., 2012). Thus, lower miR-7 level due to the sponging effect of cirS-7 causes an increase in target genes, in this instance EGFR and causes an imbalance to the signaling pathway (Fang et al., 2012).

### Several Reported CircRNAs Contribute to Treatment Resistance *via* PI3K/AKT Pathway

Aberrant fusion-circRNA (f-circRNAs), a new form of circRNA generated by chromosomal translocation, was recently linked to treatment resistance in leukemic cells. Lehmann et al. found that f-circRNAs may potentially contribute to the development of resistance to therapy by protecting the leukemic cells from arsenic trioxide (Lehmann et al., 2001). Arsenic oxide is used to treat newly diagnosed and relapsed leukemic patients where it is known to induce apoptotic and cytotoxic effects in blast cells (Dong et al., 2015). Similarly, another study found that by knocking out the expression of f-circM9, leukemic cells can resume apoptosis and resistance toward Ara-C can be reversed. The team has proved that this type of f-circRNA can contribute to tumorigenesis and resistance to therapy both *in vitro* and *in vivo.* Besides the MAPK pathway, the PI3K/AKT signaling pathway was triggered simultaneously by the presence of f-circM9. Similar to MAPK, the PI3K/AKT pathway is another common pathway often associated with cancer. This complex cascade influences cell apoptosis, cell cycle, DNA repair, glucose metabolism, and cell transformation. Mainly it is known to play a critical role in transducing signal between oncogenes and cellular functions (Gao et al., 2017). Inhibition of these two pathways caused drug induced apoptosis, hence the high presence of f-circRNAs in leukemic cells will have a negative effect on treatment response. This could be an indicator of possible treatment failure for leukemic patients (Nanba and Toyooka, 2008).

The hsa_circ_0006528 was found to play a vital role in mediating chemoresistance in breast cancer (Carrle and Bielack, 2006). It was observed that higher expression of circ_0006528 is significantly associated with adriamycin (ADM)-resistance in breast cancers (Miao et al., 2017). Circ_0006528 is derived from exons 2 to 5 of the PRELID gene and is exclusively related to miR-7-5p. Previously, it was reported that low levels of miR-7 contribute to the resistance to chemotherapy, and the findings of this study further supported this (Callaghan et al., 2014). Upregulated circRNAs triggered the MAPK signaling pathway and were shown to regulate the cancer cells response to ADM treatment (Nanba and Toyooka, 2008). PI3K/AKT could also play a part in mediating the drug resistance *via* regulation of hsa-miR-130b (Miao et al., 2017). Current evidence showed that up-regulation of miRNA-130b mediates chemoresistance and increases the proliferation of breast cancer cells. It also reduces the expression of PTEN target which is a common tumor suppressor gene responsible for cell growth and apoptosis (Di Cristofano and Pandolfi, 2000). It is worth noting that the MAPK and PI3K/AKT pathways are complex and multilayered in nature, thus we could see that different types and levels of circRNAs trigger these pathways with opposing effects. In support of this, activation of AKT pathway was shown to have a better prognosis in luminal breast cancer in contrast to its detrimental effect on the above-mentioned study involving circ_0006528 (Sonnenblick et al., 2019).

PI3K/AKT signaling pathway was also predicted to regulate response to 5-Fluorouracil (5-FU) in colorectal cancer patients. 5-FU, a pyrimidine antagonist, is widely used in managing advanced stage colorectal chemotherapy patients but has a recurrence rate of more than 50% (Yu et al., 2009). Upregulation of hsa_circ_0000504 was observed in 5-FU resistance colorectal cancer, where the circRNA interacts with miR-485-5p which targets STAT3 gene (Xiong et al., 2017). It was demonstrated that by silencing the STAT3 gene, clonogenic survival of cancer cells was significantly reduced (Spitzner et al., 2014). From the KEGG pathway analysis, miR-485-5p was predicted to activate the AKT signaling pathway which is associated with chemoradiotherapy treatment response (Xiong et al., 2017). In another recent study, it was reported that hsa_circ_0004015 was highly expressed in non-small cell lung cancer (NSCLC) which significantly contributes to disease progression and EGFR-Tyrosine Kinase Inhibitors (TKI) resistance (Zhou et al., 2019). TKIs are used as first line drug for NSCLC patients with EGFR mutations. Subsequently, *via* bioinformatics prediction, miR-1183 and target gene PDPK1 were further studied. It is known that PDPKI a crucial component of the Akt-mTOR pathway, can intervene in cell proliferation and apoptosis (Li et al., 2018). In vitro results concluded that knockdown of hsa_circ_0004015 significantly sensitized NSCLC resistant cell line to TKIs (Zhou et al., 2019). Similarly, another group of investigators also proved the relationship of circRNA in resisting EGF-TKIs treatment by activating AKT/mTOR in EGFR-mutant NSCLC (Cheng et al., 2015)

Interestingly this pathway was also seen activated in cisplatin resistance in gastric cancer (GC) (Huang et al., 2019). Cisplatin is among the main chemotherapy drugs given for GC patients. In this study circRNA AKT3 upregulates PIK3R1 which, in turn increases the treatment resistance *via* miR-198 suppression and activation of PI3K/AKT signaling pathway. Similarly, cisplatin resistance was also found in human thyroid carcinoma cells in which circRNA EIF6 was studied (Liu et al., 2018). By bioinformatics analysis, miR-144-3p was found to regulate the expression of circRNA EIF6 and Transforming growth factor-α (TGF-α). This growth factor could promote tumor growth *via* various signaling pathways, such as PI3K/AKT and MEK/VEGF. Besides, a transcription factor known as Forkhead box O (FOXO) was reported to interact with the PI3K/AKT pathway as well and is mostly targeted for cancer treatment therapy (Farhan et al., 2017). In support of this, a group of researchers has found that overexpression of circRNA 0067835 promotes the expression of FOXO3a gene which causes detrimental effect to temporal lobe epilepsy (TLE) (Guo et al., 2018). Researchers discovered that circRNA-0067835 acts as sponge to miR-155 which has binding sites to FOXO3a gene. From these findings, aberrant activation of this pathway not only contributes to the progression of disease but also the cell receptivity to chemotherapy and radiotherapy treatment (Toulany and Rodemann, 2015). This multiplex pathway consisting of many regulatory molecules are seen hyper activated in different types of cancers. Looking in a different lens, this could open a new path for research and therapy management especially in terms of chemoradioresistance.

### CircRNA Influences Uptake of Drugs *via* Drug Transporter Pathway

A group of researchers has found that downregulation of circPVT1 by small inhibiting RNA (siRNA) might weaken resistance to doxorubicin and cisplatin treatment in osteosarcoma (Kun-Peng et al., 2018). Doxorubicin or ADM is an essential component in the treatment of osteosarcoma and cisplatin is the second most commonly used drug given in combination with doxorubicin (Carrle and Bielack, 2006). This study found that cirPVT1 was significantly higher in the chemoresistance group as compared to the normal control group and knockdown of circPVT1 could partly reverse the resistance of doxorubicin and cisplatin (Kun-Peng et al., 2018). Overexpression of circPVT1 is also present in chemoresistant lung cancer as compared to chemosensitive patients. Knockdown of circPVT1 decreased the expression level of a well-known drug transporter gene, *ABCB1*. From previous discoveries, it was found that the gene promotes chemoresistance *via* removing the intracellular drugs by P-glycoprotein (P-GP) an ATP-dependent efflux pump that may have to metabolize the drugs in cancer cells (Callaghan et al., 2014).

### CircRNA Interrupt Chemotherapy Acceptance by Regulating VEGF Pathway

CircRNA has also been shown to be a potential regulator for gemcitabine resistance in pancreatic cancer (Shao et al., 2018). Gemcitabine, a pro drug used as one of the key drugs in treating pancreatic cancer is able to kill cells with active DNA synthesis and block cell cycle progression at G1/S phase (Plunkett et al., 1991; Plunkett et al., 1995). Researchers found that when chr14:101402109-101464448+ and chr4:52729603-52780244+ were silenced in gemcitabine resistant cell lines, the sensitivity towards the drug was enhanced (Shao et al., 2018). Likewise, when the circRNAs were overexpressed, the gemcitabine resistance towards the cell lines was further intensified. By further exploring the binding site for miRNA, it was shown that miR-145-5p was down-regulated in the tumor tissues and plasma of chemotherapy resistant patients (Shao et al., 2018). MiR-145 is known to be associated with gemcitabine resistance in pancreatic cancer and it is believed that ErbB and VEGF signaling biological pathways were activated (Skrypek et al., 2015; Zhou et al., 2016). The ErbB signaling pathway was reported to be involved in gemcitabine resistance in pancreatic cancer while the VEGF pathway was implicated in the progression of the disease which could also cause drug resistance (Shao et al., 2018). Not much literature is available with regards to VEGF and non-coding RNA in treatment resistance however it is worth to note that there is a link between non-coding RNAs particularly circRNAs, in tumor angiogenesis involving VEGF pathway (Abhinand et al., 2016). As pancreatic cancer is known for its poor prognosis, any avenue that could predict treatment response can ease the burden of unnecessary treatment hassle for patients. Potentially, the circRNAs can be used as biomarkers for planning the treatment of patients’ in a more personalized approach.

### CircRNA Causes Treatment Failure *via* Hypoxia-Inducible Factor-1 Regulatory Pathway

Hypoxia-inducible factor (HIF) pathway is increasingly studied due to its therapeutic potential in disease management. A group of circRNA researchers has joined the bandwagon in exploring the relationship between this pathway and cisplatin resistance in bladder cancer (Su et al., 2019). HIF 1 protein plays an integral part in angiogenesis and responding directly to hypoxia. However, due to this property, the enhancement of this gene can also allow the proliferation of cancer cells. Thus inhibition of this pathway potentially could prevent the metastasis of cancer cells (Ziello et al., 2007). In this study, circELP3 was elevated in hypoxia condition, was found to contribute to treatment resistance in bladder cancer cells (Su et al., 2019). Elevation of circELP3 was also in accordance with the severity of disease in human cancer patients. Stimulatingly, the hypoxia elevated circELP3 was independent to ELP3. In theory, it is expected that the hypoxia-related circRNA is correlated to this angiogenesis pathway however the findings in this study were contradictory. This unexpected finding gives a new perspective to the function of circRNA in regulating cisplatin resistance and further test should be carried out to confirm the independence. Another group of researchers has identified circ_103470 and circ_101102 was dysregulated in endometriosis and HIF-1 may be associated with the pathogenesis (Zhang et al., 2018). However, this was only a profiling study thus the space to explore remains large. Compared to other mentioned pathways, HIF-1 has just commenced. As angiogenesis is one of the key target approaches in cancer study, it is worth to explore this area of the pathway with a combination of circRNA.

## Future Directions and Conclusions

**Figure 1:**
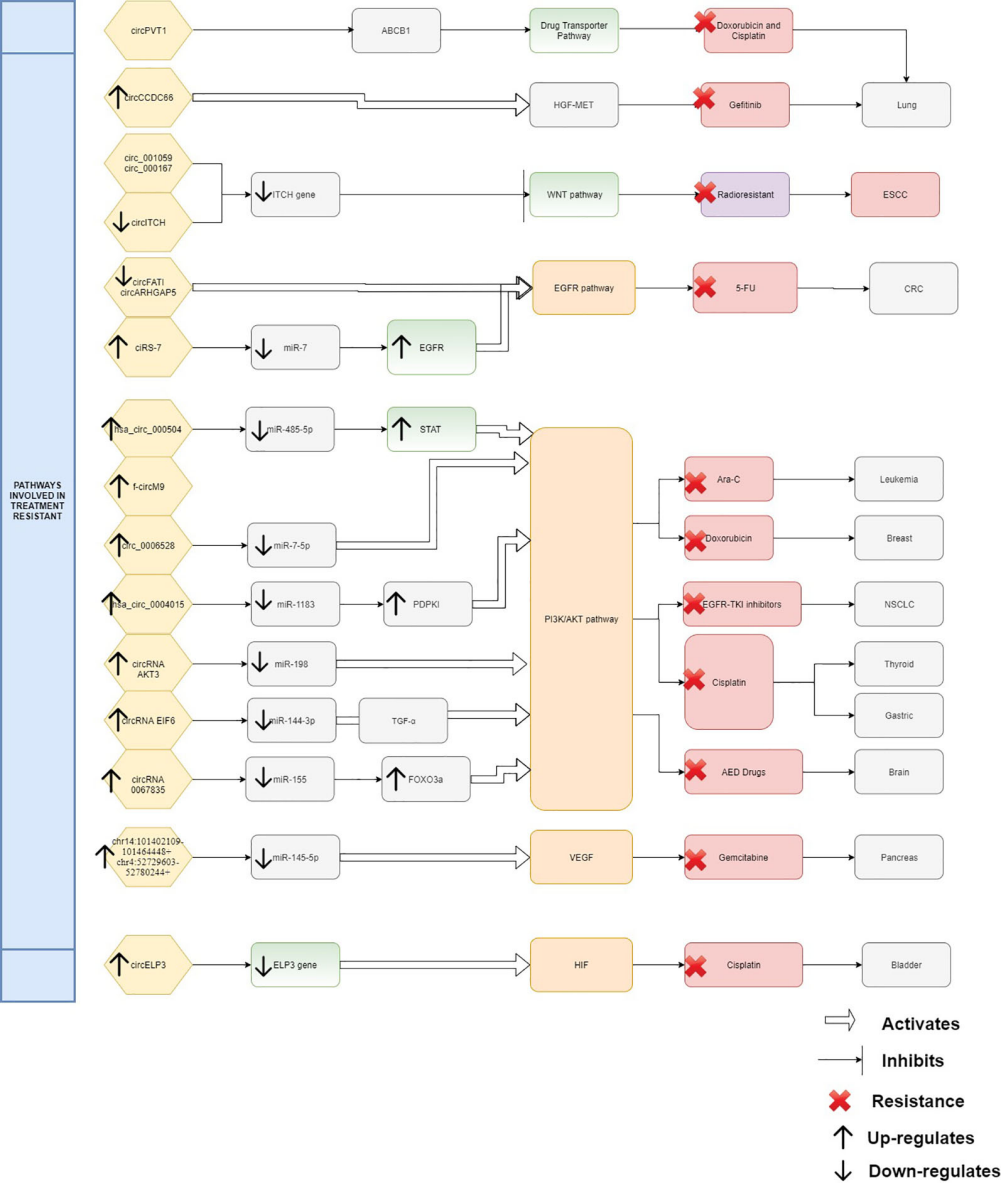
Interaction between circRNAs, targeted gene, and pathways.

From the evidence compiled as shown in **Table 1**, it was apparent that there are still many unexplored circRNAs that might be involved in cancer treatment resistance. At this juncture it is difficult to conclude the impact of circRNAs in chemoresistance and radioresistance; however, they have already exhibited enormous potential for further exploration. As treatment resistance in newly diagnosed patients or in recurrence state remains a major challenge, circRNAs could be an ideal marker to predict for treatment failure or even in reversing the resistance. Although the evidence is rather limited and incomplete, it is clear that there are certain interactions between circRNAs and miRNAs which act as contributing factors in disease progression and therapy resistance. By identifying the interactions between circRNA/miRNA/gene/pathway, more opportunities on targeted therapies can be created as shown in **Figure 1**. In this review, many circRNAs were shown to activate the MAPK pathway and the PI3K signaling pathway. By studying the complex interaction between circRNAs, targeted gene, and the pathways involved, we may be able to detect responses to treatment, early resistance or recurrence in real time. However, analyzing and targeting specific circRNAs persist as a challenge to researchers. Besides than optimizing the feature of circRNA as competing endogenous RNA, limited computational approaches, such as microarray and RNA sequencing, are used for differential expression analysis. Subsequently, inhibition and overexpression of circRNAs are carried out to functionally characterize the targeted circRNAs based on the bioinformatics analysis. These pipelines are rather complex and require tedious preparation for reliable results and remain as a great challenge for novice researchers. It is apparent that there are still gaps to be filled in terms of predicting the target genes and the functional mechanisms in modulating diseases. For validation of the high throughput sequencing, current approach mostly focuses on proving the presence of circRNA by carrying out qPCR and Sanger sequencing. To date, only a few circRNAs have been characterized and validated fully. The unpredictable character of circRNA as a pro-oncogene and anti-tumor molecule is thought-provoking and a limiting factor for immediate clinical application. It is more challenging when the same circRNA exhibit multiple roles in different cancers. Thus, it is difficult to deduce and predict a single role for circRNA. Nonetheless, this shows the flexibility of circRNAs as a biomarker and when manipulated could contribute to prevention or delaying of disease progression. CircRNAs as a predictive marker and potential regulator of treatment resistance is still in the early phase of research and application, nevertheless, the future of circRNAs is certainly promising, and it will be likely that they will become part of the armamentarium to treat cancers in future.

**Table 1:** List of CircRNAs involved in treatment resistance in human cancers.

Type of cancer	Sequence name	Expression	Intersection molecules and/pathway	Sample type	Resistant to (chemotherapy/radiotherapy)	References
Esophageal squamous cell cancer	circRNA_000167 circRNA_001059	Down	Wnt/β signaling pathway	Cell line	Radioresistant	(Su et al., 2016)
Colorectal cancer	circFAT1 circARHGAP5	Down	EGFR/MEK/ERK signaling pathway	Cell line	Chemoradioresistant	(Dou et al., 2016)
	ciRS-7	Up	miR-7/EGFR/RAF1/MAPK	Tissue	Chemoresistant	(Weng et al., 2017)
	hsa_circ_0000504	Up	miR-485-5p/AKT pathway/STAT3	Cell line	Chemoresistant (5-Fluorouracil drug)	(Xiong et al., 2017)
Lung adenocarcinoma	CCDC66	Up	HGF/c-Met pathway and MAPK	Tissue and cell line	Chemoresistant (Gefitinib drug)	(Weng et al., 2017)
Leukemia	f-circM9	Up	PI3K/AKT1	Cell line and xenograft model	Chemoresistant (Ara-C drug and arsenic oxide)	(Lehmann et al., 2001)
Osteosarcoma	circPVT1	Up	Drug transporter pathway ABCB1 gene	Tissue	Chemoresistant (Doxorubicin,cisplastin drug)	(Zeng et al., 2018)
Pancreatic cancer	chr14:101402109-101464448+ and chr4:52729603-52780244+	Up	mir-145-5p/ErbB and VEGF	Tissue and cell line	Chemoresistant (Gemcitabine drug)	(Shao et al., 2018)
Breast cancer	Circ_0006528	Up	miR-7-5p/MAPK and PI3K signaling pathway	Tissue and cell line	Chemoresistant (Doxorubicin drug)	(Nanba and Toyooka, 2008; Miao et al., 2017)
Non-small cell lung cancer (NSCLC	hsa_circ_0004015	Up	miR-1183/PDPK1/Akt-mTOR pathway	Tissue and cell line	Chemoresistant (EGFR-TKIs drug)	(Zhou et al., 2019)
Gastric cancer	circRNA AKT3	Up	miR-198/PI3K/AKT	Tissue and cell line	Chemoresistant (cisplatin drug)	(Huang et al., 2019)
Thyroid cancer	circRNA EIF6	Up	miR-144-3p/TGF-α/PI3K/AKT	Tissue and cell line	Chemoresistant (cisplatin drug)	(Liu et al., 2018)
Temporal lobe epilepsy	circRNA 0067835	Up	miR-155/FOXO3a/PI3K/AKT	Tissue and cell line	Antiepileptic drugs (AEDs)	(Guo et al., 2018)
Bladder cancer	circELP3	Up	ELP3/HIF)-1	Tissue and cell line	Chemoresistant (cisplatin drug)	(Su et al., 2019)

## Author Contributions

SJ and NA drafted the article. NA conceived the idea. NM, EH, and RJ provided feedback and critical input.

## Funding

This manuscript was funded by the Fundamental Research Grant Scheme (FRGS/1/2017/SKK08/UKM/03/3) awarded by the Ministry of Higher Education Malaysia.

## Conflict of Interest

The authors declare that the research was conducted in the absence of any commercial or financial relationships that could be construed as a potential conflict of interest.
